# 

**Published:** 1906-06-16

**Authors:** 


### THE PREPARATION OF PALATINOIDS.

Messrs. Oppenheimer, Son and Co. write under date of the 5th instant as follows:—

'Since at the present time there is very considerable agitation regarding the necessity for the strictest scientific supervision of all animal products, and there is existent among the medical profession a certain hesitation as to whether glands and other substances from animals can be thoroughly relied upon as being in a perfectly healthy and suitable condition for employment as remedies in the treatment of diseases, we beg to inform you that all thyroid glands, suprarenal glands, and other parts of animals used in medicine and put up in the form of palatinoids, are exclusively obtained from the London abbatoirs, where the strictest Government supervision always prevails. Moreover, we have the advantage of using these in a perfectly fresh condition, so that they are worked up each day after the animals are slaughtered, and being hermetically sealed up in the palatinoids, they cannot suffer from deterioration or decomposition owing to exposure to the air.

### Medical Appointments and Vacancies.

### APPOINTMENTS.

Dr. John Phillips, M.H., M.P., F.R.C.P., has, on retirement from the post of physician, been elected consulting physician to the British Lying-in Hospital. · Dr. R. Hamilton Bell, M.A., M.D., F.R.C.P., has been elected physician to out-patients.

### VACANCIES.

Blackburn and East Lancashire Infirmary.—Junior House Surgeon.
Bolton Infirmary and Dispensary.—Junior House Surgeon.
Brighton Throat and Ear Hospital, Church Street, Queen's Road.—Non-resident House Surgeon.
Egyptian Government, Ministry of Education.—Professor of Midwifery and Gynæcology. Also Medical Tutor and Registrar to Kasr-el-Ainy Hospital.
Evelina Hospital for Sick Children, Southwark, S.E.—Assistant House Surgeon. Also Radiographer. Also Clinical Assistants.
Grimsby and District Hospital.—Resident House Surgeon.
Hospital for Diseases of the Throat, Golden Square, W.—Honorary Assistant Surgeon.
Italian Hospital, Queen Square, London, W.C.—House Surgeon.
Leeds, City of, Sanitary Committee.—Assistant Medical Officer of Health and Chief Inspector of Nuisances.
Liverpool Hospital for Consumption and Diseases of the Chest.—Pathologist and Assistant to the Medical Staff.
London Hospital, Whitechapel, E.—Medical Registrarship.
New Hospital for Women, Euston Road, London.—Ophthalmic Surgeon (female).
Northampton General Hospital.—Assistant House Surgeon.
Royal London Ophthalmic Hospital, City Road, E.C.—Refraction Assistant.
Stirling District Asylum, Larbert, N.B.—Assistant Medical Officer (female).
West London Hospital, Hammersmith Road, W.—House Physician.
Wokingham, Berks, London, Open-air Sanatorium.—Assistant Medical Officer.
Wrexham, Borough of.—Medical Officer of Health.

### MARRIAGE.

**Notices of Births, Deaths, and Marriages are inserted in this column at a charge of 2s. 6d. for an announcement not exceeding four lines.**

BOYLE—WOOLL.—On June 9th, at St. Katharine's, Southbourne, by the Vicar, the Rev. Philip Johnstone, M.A., Edward Seymour Skinner Boyle, eldest son of Commander E. B. Boyle, R.N., of Southbourne, to Florence, daughter of William Wooll, Esq., of Cambridge.

### SUCCESS! at the C.M.B.

Examinations can only be achieved by acquiring a thorough Theoretical and Practical Knowledge of the subject.

### A COMPLETE HANDBOOK OF MIDWIFERY,

**By Dr. J. K. WATSON,**

has been designed to meet the requirements of the Central Midwives Board, and will prove of the greatest assistance to all Nurses entering for the next Examination.

6/- net, 6/4 post free.

Profusely Illustrated, with 143 Diagrams, &c.

### SIMPLE INTRODUCTORY LESSONS IN MIDWIFERY.

**By M. LOANE,**

Author of "Outlines of Routine in District Nursing."

1/- net, 1/1 post free.

This work is an easy introduction to the theory of Midwifery, for the use of those who desire ultimately to pass the Central Midwives Board Examination, and who have some practical experience of midwifery, but who are quite ignorant of the theory, and so completely unacquainted with the language in which it is usually set forth, that they are unable to profit immediately by any ordinary courses of lectures on the subject.

***JUST PUBLISHED.***

The following new Book will be found of the greatest value to Midwives and Maternity Nurses.

### THE CARE OF CHILDREN:

**Practical Hints for Mothers and Nurses at Home and Abroad.**

BY

**ROBT. J. BLACKHAM, D.Ph. (Lond.).**

1/6 net, 1/8 post free.

Contains:—Hints on Food, Drink, and Medicines, Clothing, Cleanliness, Rest and Exercise, Infectious Diseases, on the Nursery, on Nursery "First Aid," on Nursery Cooking, &c. &c.

**The Scientific Press, Ltd.,**

*Catalogue of Nursing Manuals sent post free on application.*

**Publishers of Nursing Text-Books, Charts, and Case-Books of all Descriptions.**

**28 & 29 SOUTHAMPTON STREET, STRAND, LONDON, W.C.**

